# Microstructure and Properties of Binderless μWC Obtained Using the Electroconsolidation Method

**DOI:** 10.3390/ma18204646

**Published:** 2025-10-10

**Authors:** Edvin Hevorkian, Waldemar Samociuk, Miroslaw Rucki, Zbigniew Krzysiak, Daniel Pieniak, Volodymyr Nerubatskyi, Volodymyr Chyshkala, Serhii Lytovchenko, Leszek Chalko, Dmitrij Morozow, Jacek Caban, Vitalii Kulich

**Affiliations:** 1 Department of Mechanical Engineering and Automation, Faculty of Production Engineering, University of Life Sciences in Lublin, 28 Głęboka St., 20-612 Lublin, Poland; edsgev@gmail.com (E.H.); zbigniew.krzysiak@wp.pl (Z.K.); 2 Institute of Mechanical Science, Vilnius Gediminas Technical University, 11 Sauletekio Al., LT-10223 Vilnius, Lithuania; 3 Institute of Sustainable Technologies, Lukasiewicz Research Network, 6/10 Pułaskiego, 26-600 Radom, Poland; daniel.pieniak@itee.lukasiewicz.gov.pl; 4 Department of Electrical Energetics, Electrical Engineering and Electromechanics, Ukraine State University of Railway Transport, 7 Feuerbach Sq., 61050 Kharkiv, Ukraine; 5 Department of Reactor Engineering Materials and Physical Technologies, V.N. Karazin Kharkiv National University, 4 Svobody Sq., 61022 Kharkiv, Ukraine; 6 Faculty of Mechanical Engineering, Casimir Pulaski Radom University, 54 Stasieckiego, 26-600 Radom, Poland; leszek.chalko@urad.edu.pl (L.C.); d.morozow@uthrad.pl (D.M.); 7 Faculty of Mechanical Engineering, Lublin University of Technology, Nadbystrzycka 36, 20-618 Lublin, Poland; j.caban@pollub.pl; 8 V. Bakul Institute for Superhard Materials, National Academy of Science of Ukraine, 2 Avtozavodska Str., 04074 Kyiv, Ukraine

**Keywords:** composites, nanopowder, sintering, electroconsolidation, tungsten carbide, nanocomposite, ceramic matrix composite

## Abstract

This paper contributes to the knowledge of binderless tungsten carbide (WC), which attracts the attention of many engineers and scientists for its superior properties, but its application is limited due to difficulties with the consolidation of initial powders. In the present study, the microstructure and mechanical properties of binderless WC, sintered with the electroconsolidation technique from the initial powder of a grain size of 100–200 nm, were investigated. The material was compared with nWC sintered with the same method from a nanopowder with particles of size ca. 70 nm. The binderless μWC demonstrated hardness of *HV* = 30.06 ± 0.09 GPa, which is almost 14% higher than that of nWC, but its fracture toughness was lower (*K_IC_* = 6.59 ± 0.46 MPa·m^1/2^ under 1 kg load). These differences can be attributed to the improved homogeneity of the μWC microstructure, where no large agglomerates appeared to be present in nWC. The measured plastic properties, with no signs of brittle fracture, further confirm the applicability of the binderless WC under contact stress conditions.

## 1. Introduction

Tungsten carbide WC is among the most widely used industrial hard materials [1]. It is known for its wear resistance and temperature stability [2]. Traditionally, tungsten carbide is used in the form of hard alloys with a metal matrix binder, usually cobalt [3,4,5]. WC-based materials have proven to be feasible for cutting tools destined for the machining of various metal alloys. Among the main advantages of these materials is the advantageous balance between hardness and toughness. Tungsten carbide is perhaps one of the most popular materials, but unfortunately, its main ingredients, W and binder Co, are at high risk in terms of resource supply, and they are listed in EU documents among critical raw materials (CRMs), demanding sustainable use [6].

Currently, a large number of grades of hard alloy materials are available on the market and are being investigated. They differ from one another by the percentage of the binding phase [7,8]. However, the presence of cobalt or other binding components has a number of disadvantages. First of all, cobalt may undergo corrosion and thermal degradation, which reduces the service life of products and poses a limitation to operation in high-temperature conditions. Secondly, the properties of a metal binder determine the reduced maximum achievable values of hardness and heat resistance of the material. Moreover, from an environmental perspective, the presence of cobalt is undesirable due to its toxicity and possible carcinogenic effects [9,10]. The presence of cobalt in cemented carbide makes it virtually impossible to recycle worn-out components. At the same time, the acquisition of recycled tungsten from end-of-life (EoL) components is becoming increasingly important. According to the International Tungsten Industry Association (ITIA), the global tungsten flow indicated that the total input for production of intermediates was 108,500 metric tons of tungsten content with a recycling input rate of 35%, while the end-of-life recycling rate can be calculated as 30% [11]. Thus, binderless tungsten carbide without any addition of metallic components is attracting increasing attention from scientists and engineers [12,13,14].

Densification of tungsten carbide without a metal binder is a challenging and technologically complex process that requires proper material design in order to solve the double problem of densification and brittleness [15]. It is possible to use traditional powder metallurgy methods like sintering in vacuum or inert gas at temperatures above 2000 °C under the application of uniaxial pressure. Without a binder, sintered WC requires more precise control of the residual oxygen level and holding time, to avoid formation of W_2_C and other undesirable phases. The first patented tungsten carbide material without a metal binder was likely proposed by Kodash and Gevorkyan [16], along with the respective hot-pressing technology.

The development of ultra-dispersed powder synthesis and of high-temperature powder metallurgy techniques has made it possible to fabricate dense, monolithic structures of tungsten carbide without any metallic binder. This opened up prospects for creation of new-generation super-strong, corrosion- and heat-resistant materials. Using commercially available submicron WC powder, Gubernat with colleagues [17] obtained binderless WC polycrystals of 96–98% relative density, exhibiting high fracture toughness between 7 and 11 MPa∙m^0.5^, as well as bending strength of ca. 0.8 GPa, regardless of introduced additives. A very small initial particle size contributed to the successful sintering of binderless WC. Typically, the used powders were of particle sizes less than 1 µm, but best results were obtained with particles of ca. 50–100 nm [18]. It was found that the smaller grain sizes required lower sintering temperatures to achieve the full density and elevated final hardness of the material, due to the Hall–Petch strengthening mechanism [19,20]. However, commercial WC nanopowder with a particle size of 200 nm is ca. two times more expensive than that of ca. 2 μm. Thus, in the present study, we focused on the possibility of achieving comparable characteristics of binderless WC sintered from initial micropowder instead of nanopowder.

One of the most effective technologies for the fabrication of binderless WC compacts is Spark Plasma Sintering (SPS), also known as the Field-Activated Sintering Technique (FAST). In this process, a high-density current passes through the powder batch, causing a localized rise in temperature and ensuring rapid sintering of the particles [21]. This method allows for obtaining a high density at relatively low sintering temperatures and short holding times, which prevents excessive grain growth [22]. Silvestroni et al. reported on the possibility of fabricating binderless WC, which retained high strength and toughness in the temperatures up to 1500 °C [23]. Qin and co-authors demonstrated the remarkable corrosion resistance of the binderless WC-based composite in an alkaline environment [24]. However, when it comes to sintering WC powders with a particle size around 4 μm, at a holding time over 1 min and a sintering temperature over 1700 °C, Cha and Hong reported abnormal grain growth [25]. Among technological difficulties, Dopita et al. [26] indicated the fact that the agglomeration of tungsten carbide nanopowders might reduce the strength and toughness of the material.

In the present study, a novel, modified SPS technique was applied. It was called electroconsolidation to emphasize the role of electrical current in the densification and consolidation processes [27]. Compared to traditional SPS, high AC is applied directly to the powder compact, without any pulse generator, which significantly reduces the cost. Using this technique, binderless WC was sintered from powders with ca. micron particle size, achieving comparable properties to that of nanodispersed WC. Thus, the novelty of this work covers both the electroconsolidation technique being a cheaper and resource-saving alternative to classical SPS methods, and the possibility of fabricating binderless WC compacts using microdispersed initial powders instead of nanopowders.

## 2. Materials and Methods

**Figure 1:**
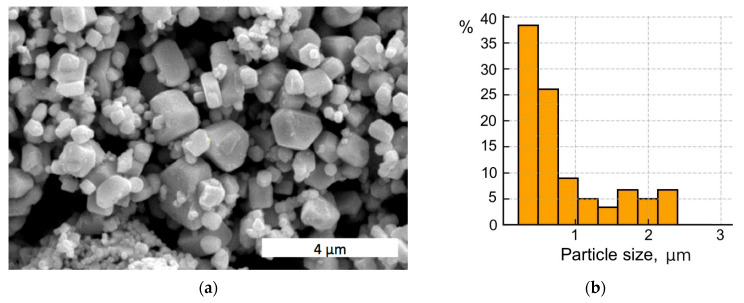
Initial WC powder: (**a**) SEM image; (**b**) particle size distribution.

In the research, ultrafine powder of tungsten carbide with submicron average particle sizes was used (Pol-Aura, Zabrze, Poland). As can be seen in Figure 1a, some larger particles of dimensions close to 1 μm are present, along with agglomerates of nano-sized particles. A histogram of the WC particle size distribution is presented in Figure 1b.

**Figure 2:**
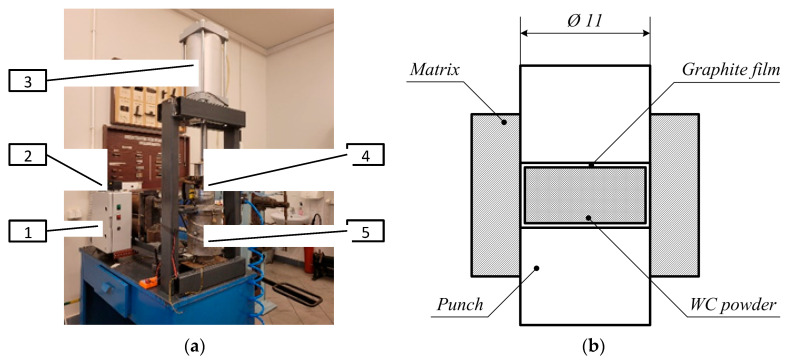
Preparation of the sintered binderless μWC samples: (**a**) apparatus for electroconsolidation; (**b**) preliminary pressing of the sample in a metal mold. 1—power block, 2—current and temperature control unit, 3—pneumatic actuator for uniaxial pressure, 4—built-in graphite mold, 5—vacuum chamber.

Sintering was carried out using the patented electroconsolidation device [27], described in detail in [28,29]. Its essential distinguishing feature is the industrial alternating current applied directly to the sintered powder under uniaxial pressure, without any sort of pulse generator, usually applied in SPS. A general view of the unit is shown in Figure 2a, with the main components denoted as follows: (1) power block, (2) current and temperature control unit, (3) pneumatic actuator for uniaxial pressure, (4) built-in graphite mold, (5) vacuum chamber. The vacuum chamber had a volume of 7 dm^3^ and allowed for obtaining pressure of 10^–3^ Pa. In the vacuum chamber, a conductive graphite press mold is placed between the current leads.

In order to ensure good densification, the powder underwent preliminary pressing in a metal mold with a thin graphite interlayer between the mold and the sample. This operation is shown in Figure 2b. The diameter of the punch was 11 mm, and the pressure was 50 MPa.

Before pre-pressing, the inner surface of the metal mold was covered with a graphite film. After the procedure, a thin layer of graphite remained on the surface of the sample. The sample was then placed into a graphite mold for further hot pressing and electroconsolidation. Heating was carried out up to 1000 °C at a rate of 150 °C/min, while the pressure was gradually increased from 20 MPa up to 45 MPa. After reaching 1000 °C, the heating continued at a rate of 250 °C/min until the sintering temperature was reached. In order to assess the effect of processing temperature, the samples were prepared at different sintering temperatures: 1300 °C, 1400 °C, 1500 °C, 1600 °C, and 1700 °C. The holding time was 2 and 3 min for each temperature to assess its effect on the densification process and phase evolution. Cylindrical compacts with a diameter of 11 mm were produced, and their typical thickness after sintering was 3–4 mm. Before the analysis, the sintered specimens were ground and polished, obtaining the submicron roughness shown in Table 1. The polishing procedure with silver discs and water coolant lasted 3 h 20 min, since shorter durations left some visible directional scratches.

The surface of the samples was examined using a Hitachi SU-70 field emission scanning electron microscope (FE-SEM) (Hitachi, Krefeld, Germany) equipped with an electron gun with a Schottky thermal emitter. A Thermo Scientific (Waltham, MA, USA) EDS-type X-ray microanalyzer was used, which allowed for qualitative and quantitative analysis through the detection of various elements by acquiring an X-ray spectrum depending on the energy characteristics of the radiation of a given element. Phase analysis of the initial powder and sintered samples was performed using Cu Kα radiation (λ ≈ 1.5406 Å), 2θ range 20–90°, step 0.02°, with sufficient counting statistics. XRD patterns were processed using the Rietveld refinement method to quantify the WC, W_2_C, and W phase fractions.

Fracture toughness was determined using a diamond Vickers pyramid indenter under a load of 49 N. Crack propagation initiated from the corners of the indentation allowed for determination of the stress intensity factor, with its critical value known as the fracture toughness *K_IC_* of the material. This is known as the first case of Griffith cracking, a crack in a specific loading conditions. To determine the *K_IC_*, the crack length *c* and the diagonal dimension of the indentation *a* were measured. Then, toughness was calculated using the following commonly known equation [30]:

(1) $$K_{IC}=0.067\cdot {\left(\frac{E}{HV}\right)}^{0.4}\cdot {\left(\frac{c}{a}\right)}^{-1.5}\cdot HV\cdot \sqrt{a},$$

where *a* is half of the diagonal of the imprint, m; *E* is the modulus, GPa; *HV* is hardness, GPa; *c* is the crack length, m.

Since the samples of the powder contained agglomerates, the microindentation test was performed according to the standard ISO 14577-1:2015 [31] in order to evaluate the mechanical properties of the matrix and separately those of dispersed grains (inclusions). The Hysitron TS77 Select device produced by Bruker (Billerica, MA, USA) was used to evaluate the characteristics of the material phases. The in situ SPM imaging mode was used for a topographic survey of the surface. It was performed using a Berkovich indenter under a load of 10 mN. Its projected contact area was *A* = 24.5 *h_c_*^2^, a function of the depth *h_c_*. The micromechanical properties, including hardness at each indentation point, were simultaneously determined according to the following equation [32]:

(2) $$H_{IT}=\frac{P_{max}}{A},$$

where the indentation hardness *H_IT_* was the ratio of the largest normal loading force *P_max_* to the indenter contact area *A* at maximum load.

Additionally, scratch tests were performed according to ASTM G171-24 [33]. The permanent scratch depth (*R_d_*) was determined as a critical parameter characterizing the resistance of the material surface to mechanical damage caused by a moving indenter under an applied load. This parameter reflects the extent of plastic and, in some cases, brittle deformation resulting from contact stresses during scratching. The samples were tested on a Micro Scratch Tester (MST, Anton Paar, GmbH, Ostfildern, Germany). First, one surface of the samples was processed on a laboratory grinder–polisher ATM Saphir 550 (ATM GmbH, Blieskastel, Germany). Then, scratches were made on this surface using a Rockwell indenter in the form of a diamond cone with a rounding radius of 100 µm. The test load, whose vector was perpendicular to the tested surface, was varied in 4 incremental steps, increasing from 10 N up to 20 N in successive stages. The scratch test was conducted at a speed of 2.5 mm/min, resulting in a total scratch length of 1 mm. The shape and geometric dimensions of the scratch were assessed microscopically using the microscope, which is an integral part of the MST device.

## 3. Results and Discussion

### 3.1. Elemental and Phase Composition

**Figure 3:**
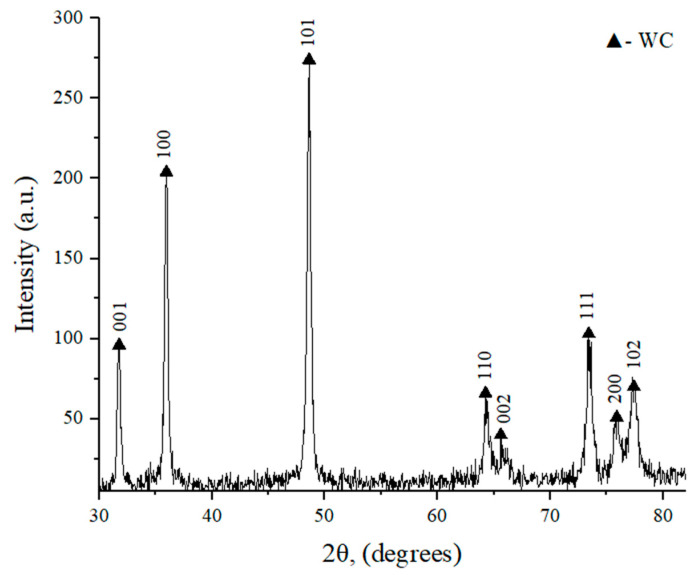
XRD pattern of the initial WC powder (100–200 nm).

**Figure 4:**
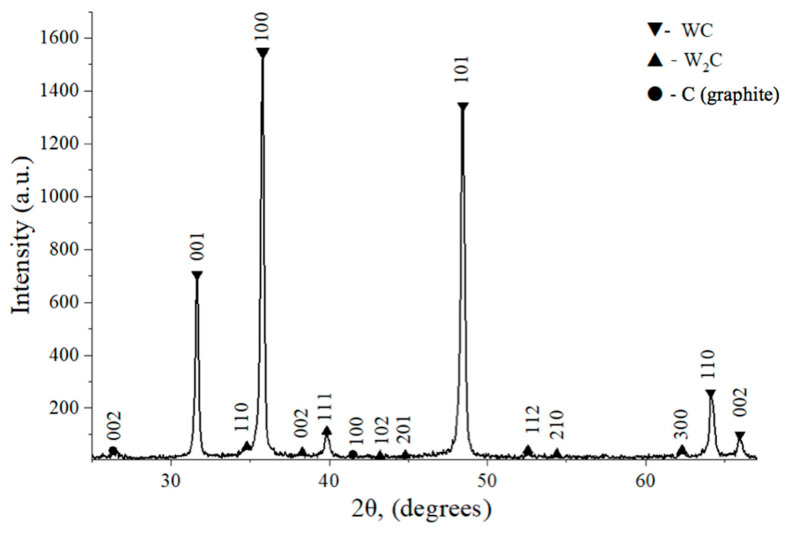
XRD pattern of the binderless WC sintered at *T* = 1700 °C and *p* = 45 MPa, holding time of 2 min.

Results of an XRD spectral analysis of the studied samples before and after sintering are shown in Figure 3 and Figure 4, respectively. The quantitative results of two measurements are collected in Table 2.

It is worth noting that the lattice parameters of the WC phase remained unchanged after sintering. Higher temperatures (above 1500 °C) favor partial decomposition of WC into W_2_C and C, as indicated in Table 2. A significantly high content of the brittle W_2_C phase contributes to the reduced strength of the material and its increased hardness. Sintering in vacuum or carbon-deficient conditions (e.g., due to oxidation of carbon or carbon loss to reaction with residual oxygen) can lead to WC decomposition via the intermediate W_2_C phase:

2WC → W_2_C + C, followed by (3)

W_2_C → 2WC + C. (4)

In order to improve our understanding of these phenomena, additional phase analysis was performed to compare the quantities of WC, C, and W_2_C in specimens sintered at different temperatures. The results are presented in Table 3.

Data in Table 3 demonstrate that below 1500 °C, sintered structures consisted mainly of WC, without significant amounts of side compounds. However, at temperatures of 1600 °C and higher, significant percentage of W_2_C appeared, together with respective amounts of free carbon. Thus, the reaction described by Equation (3) took place at elevated temperatures and could not be avoided even with high heating rates. The appearance of small amounts of W_2_C is unavoidable when sintering binderless WC compacts due to the surface oxidization of the WC powder in air.

The presence of W_2_C in the sintered WC always affects its mechanical characteristics [34]. The obtained XRD data correlates with microstructural features and properties, in particular, an increased percentage of W_2_C contributed to enhanced hardness but decreased fracture toughness. These phenomena are responsible for the phase compositions and related mechanical properties obtained at different sintering temperatures.

**Figure 5:**
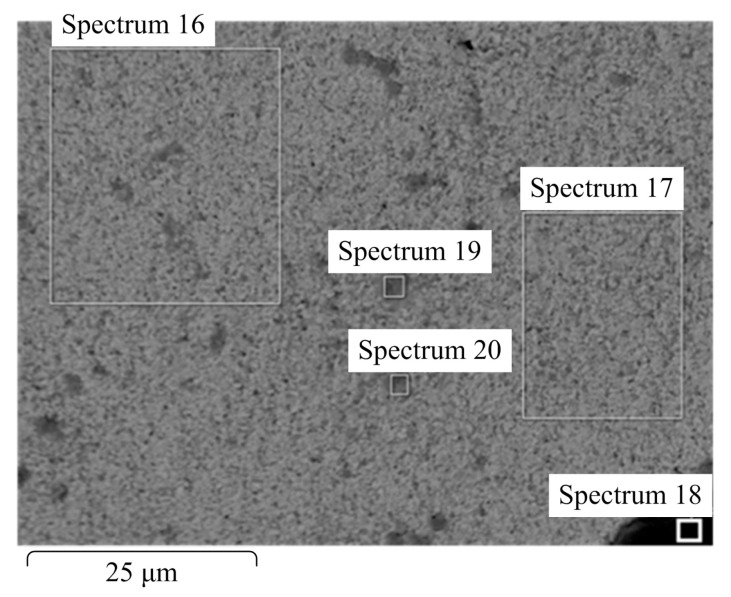
Areas chosen for further elemental analysis of the binderless WC obtained with electroconsolidation at *T* = 1700 °C and *p* = 45 MPa, holding time of 2 min.

**Figure 6:**
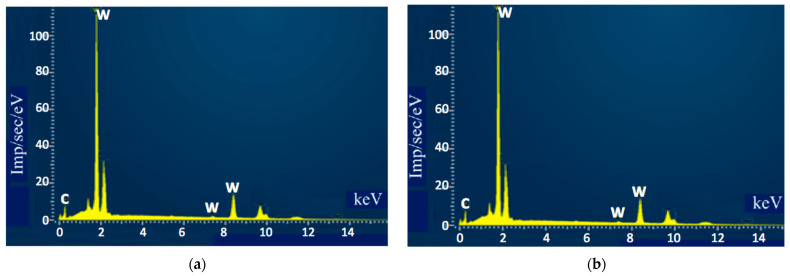
Qualitative elemental analysis of the sample surface: (**a**) Spectrum 16 marked in Figure 5; (**b**) Spectrum 17 marked in Figure 5.

The microstructures of the sintered samples appeared to be uneven, with some irregular darker areas with dimensions of a few microns, as seen in Figure 5. To obtain insight into the elemental composition of different grains, analysis of the marked areas that contained different numbers of darker inclusions was carried out. The results are shown in Figure 6 and in Table 4.

Despite a significant visual difference between areas 16 and 17, the respective spectra revealed essentially the same results. Therefore, it can be assumed that the darker places in area 16 indicate concentrations of grains with different sizes, most likely emerging in places where agglomerates of nanoparticles were located. On the other hand, the small dark areas marked 18, 19, and 20 appeared to contain impurities in large overall amounts (close to 20%).

**Figure 7:**
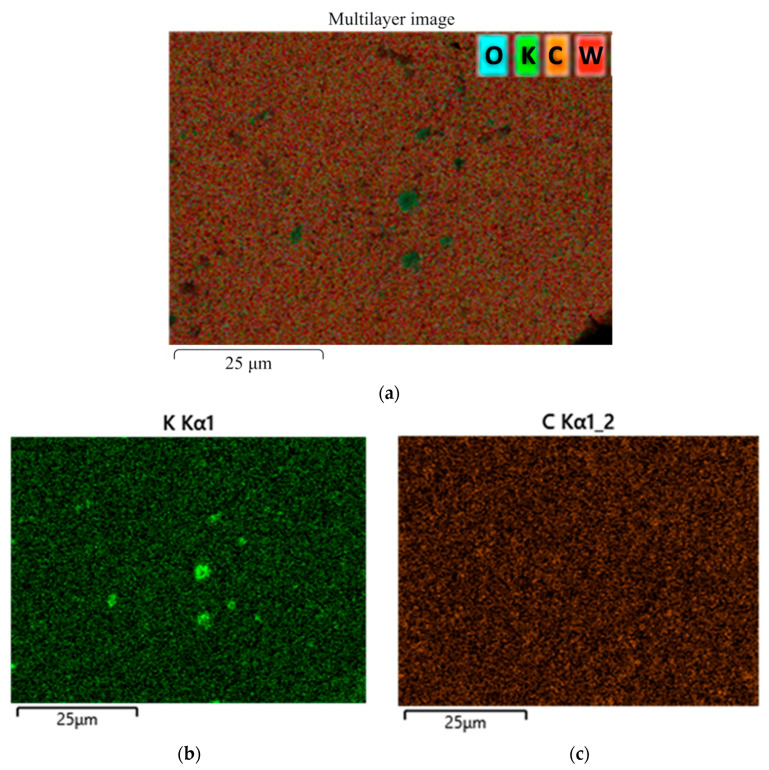

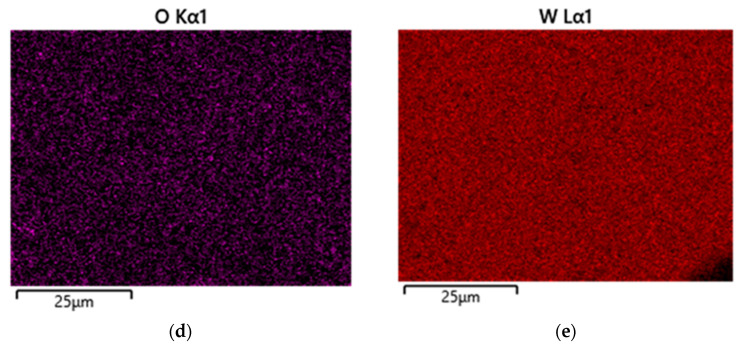
Microfluorescence analysis of the distribution of chemical elements on the surface of a sintered sample: (**a**) multilayer map of the elements; (**b**) map of the potassium distribution; (**c**) map of the carbon distribution; (**d**) map of the oxygen distribution; (**e**) map of the tungsten distribution.

Additional analysis proved that the detected Ca, Na, K, and Cl impurities did not originate from the sintering process and were present in the powder batch only at trace levels below 0.2 wt.%. Similarly, the mean concentrations of these elements in the sintered ceramics were determined to be in the range of 0.1–0.3 wt.%. The most likely sources include, for example, residuals from chemical synthesis, contamination during powder handling, storage, or transport, and wear products from milling/grinding media. It should be noted, however, that the impurities appeared locally in several spectra and were absent in homogenous areas of the sintered specimens. Elemental distribution maps shown in Figure 7 indicate that impurities, e.g., potassium, tended to concentrate in limited areas, while other elements were distributed steadily. Due to the presence of the inclusions of impurities, additional attention was paid to the characteristics, but no significant impact on the measured hardness or toughness was found. Perhaps a larger amount of these impurities or their even distribution throughout the specimen would have affected the properties, but our experimental results demonstrate their negligible effect.

### 3.2. Microstructure and Physical Properties

**Figure 8:**
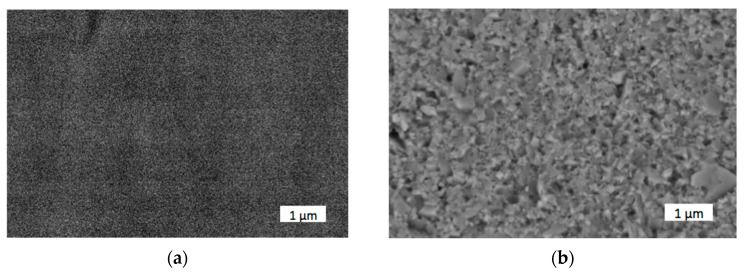
Microstructure of fracture surface of binderless WC sintered at 1700 °C and held for 2 min: (**a**) initial powder was 100–200 nm (μWC); (**b**) initial powder was 50–70 nm (nWC).

High magnification images obtained in backscattered electron (BSE) mode, as shown in Figure 8, exhibit dense grain compaction (close grain connection without clear boundaries), indicating a sufficient degree of sintering. This is especially important considering the challenging sinterability of binderless tungsten carbide [35].

However, from images in Figure 5, it is not possible to determine the shape of the grains because their boundaries are not pronounced. The images show uniform contrast, without lighter or darker inclusions, confirming the identical chemical nature of all grains—pure WC. The absence of contrast with the binding phase (e.g., cobalt), which would appear lighter in BSE, confirms that the sample was indeed free of metal bonding. No pores are visible in the images, indicating a high degree of compaction. No microcracks were detected either; therefore, the structures appear mechanically stable after sintering.

**Figure 9:**
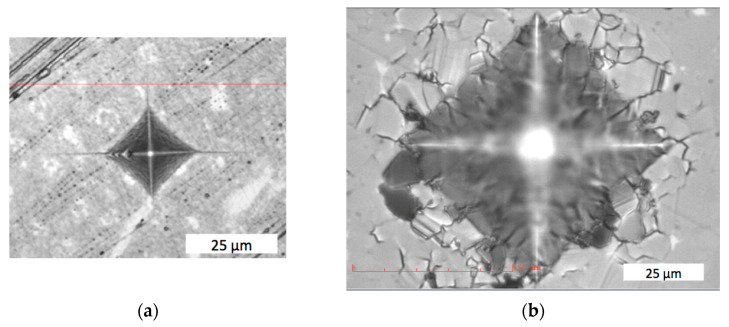
Crack propagation after indentation: (**a**) binderless μWC sintered at *T_s_* = 1700 °C with holding time of 3 min; (**b**) WC–6 wt.% Co composite sintered at *T_s_* = 1350 °C with holding time of 3 min.

After an indenter was applied to the binderless μWC material, cracks appeared at the corners of the imprint and were almost linear, directed outwards from the center, as shown in Figure 9a. In contrast, in the WC–Co system, cobalt binder appeared to be the weak element, resulting in intergranular damage around the imprint, as seen in Figure 9b.

The crack propagation in the binderless μWC corresponds with the Griffith cracking model; therefore, Equation (1) can be applied for fracture toughness calculation. Based on the applied load, the hardness and fracture toughness were measured, as follows: *HV*_5_ = 28.95 ± 0.34 GPa and *K_1C_* = 5.4 ± 0.43 MPa∙m^0.5^ under load 49 N; and *HV* = 30.06 ± 0.09 GPa and *K_1C_* = 6.59 ± 0.46 MPa∙m^0.5^ under load 9.8 N. Table 5 presents comparison of mechanical properties of WC–Co composite with binderless μWC and nWC sintered materials. Importantly, the standard, commercially available WC–8 wt.%Co composition has been sintered at a lower temperature, but for 60 min, which results in much higher energy consumption.

The high fracture toughness of the WC–Co systems can be attributed to crack deflection and branching seen around the indentation in Figure 9b, which increased energy dissipation and improved the toughening mechanism [37]. On the other hand, nWC exhibited fracture toughness *K_IC_* = 8.5 ± 0.5 MPa∙m^1/2^, overlapping with that of WC–6Co *K_IC_* = 9.0 ± 0.4 MPa∙m^1/2^, while its hardness was higher by 70% and reached 26.4 ± 0.5 GPa.

As for μWC properties, compared to those of nWC, an increase in hardness by ca. 14% and a decrease in fracture toughness by ca. 22% was noted. According to the Hall–Petch relationship, the main feature that contributed to this difference is the grain size [38], which is almost 10 times larger in μWC than in nWC material. An increase in hardness may be considered desirable in many applications, and since the sintering parameters and the respective energy consumption are comparable, the significantly cheaper initial micropowder substantially decreases the overall fabrication costs of binderless WC.

**Figure 10:**
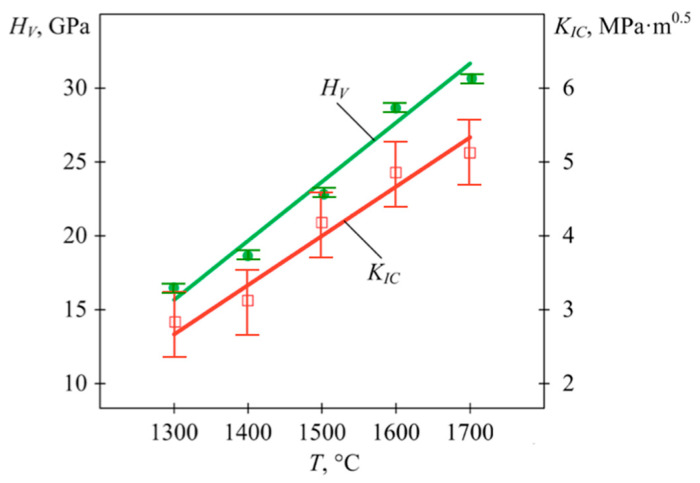
Dependence of the mechanical characteristics of μWC on sintering temperature.

The properties of μWC, as shown in Table 5, were obtained at a sintering temperature of 1700 °C. Lower temperatures provided worse results both for hardness and for fracture toughness. The dependence of these characteristics on the sintering temperature was close to a proportional function, as can be seen in Figure 10. An increase in temperature from 1300 °C up to 1700 °C caused an almost twofold increase in *HV* and *K_IC_*. The observed increase in hardness and toughness obtained at higher sintering temperatures can be attributed to the improved densification, and thus the reduced porosity, stronger intergranular bonding, and more uniform grain growth.

For further microstructural investigation and scratch tests, the specimens sintered at 1700 °C were chosen. They exhibited the highest relative density of 99.0%, as well as the highest hardness and fracture toughness. As such, they were found to be the most suitable for further detailed examinations.

**Figure 11:**
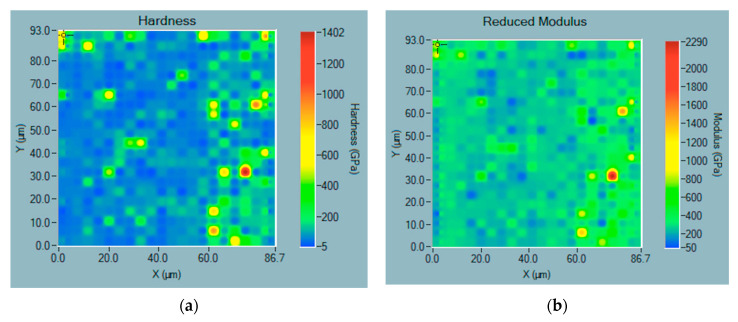
Nanoindentation mapping obtained from the Bruker Hysitron system for μWC sintered at *T* = 1700 °C for 3 min: (**a**) hardness; (**b**) reduced modulus.

Nanoindentation mapping allowed for assessment of the hardness and modulus distribution across the material surface. An example of an obtained map for μWC sintered at *T* = 1700 °C for 3 min is shown in Figure 11. It should be noted that in the measured area, there are points of significantly higher hardness and modulus.

Significant differences in hardness between nanoindentations can be associated with the orientation of WC grains, which exhibit significant variation in hardness depending on crystal orientation, as reported in many papers, e.g., [39,40]. In addition, presence of the W_2_C phase may significantly alter the properties of single points, since W_2_C may exhibit much higher hardness than WC [41]. Other researchers reported a significant improvement in microhardness of the WC–W_2_C composites compared to that of a pure WC sample [42]. Thus, the presence of areas with increased hardness could be expected due to the formation of W_2_C phase in the WC compacts (Table 3). It is worth noting, however, that such local points of increased hardness had no significant effect on the macroscopic average characteristics. Statistical distribution of the indentation results did not reveal substantial decrease in microhardness in other areas. In particular, no areas with worsened properties that might be attributed to grains with impurities indicated in Table 4 were found. Overall, the mechanical performance of the material remained consistent.

### 3.3. Scratch Resistance

**Figure 12:**
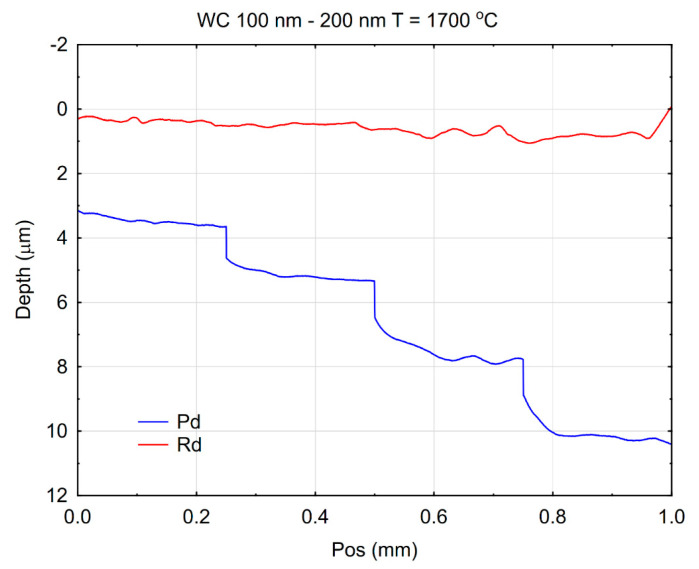
Penetration depth of the indenter under load (Pd) and the residual (permanent) scratch depth (Rd) of binderless μWC sintered at 1700 °C.

The scratch wear resistance, represented by the depth of surface damage on the tested material, was quantitatively evaluated and is presented in Figure 12. The sample was obtained by electroconsolidation at 1700 °C.

A scratch with a pointed element resulted from the combined action of forces normal and tangential to the surface [43]. Scratches can also result from the impact of small particles on the surface [44]. For many engineering materials, surface scratch tests consist of linear movement of an indenter loaded with a constant or increasing normal force. In this study, the increasing force method was used. The experimental results revealed a distinct load-dependent behavior. When a normal load of 1 N was applied to the indenter, the depth was the lowest. However, with a load of 10 N, the highest depth was reached. This dependence is seen in the clearly stepped penetration depth curve, denoted Pd and marked in blue in Figure 12. However, such behavior under the load does not imply permanent damage. The permanent scratch depth varied much less with the indenter load, which is represented by the curve denoted Rd and marked red in Figure 12. This result may indicate a significant contribution from elastic load, influencing the course of the Pd curve. This suggests that under relatively low loading conditions, the μWC surface offered less resistance to indentation-induced deformation. The phenomenon may be attributed to microstructural factors, such as grain boundary characteristics, porosity, or surface roughness, which can influence the initial material response. The enhanced performance under elevated loads can be explained by the intrinsic properties of tungsten carbide—namely, its extremely high hardness, strong covalent bonding, and resistance to plastic flow and fracture under compressive stress. The transition in scratch resistance behavior with increasing load reflects the complex mechanical response of WC-based materials. While more prone to surface indentation, they benefit from high structural rigidity and fracture toughness at elevated stresses, which likely leads to reduced progression of the scratch depth. Such results are particularly relevant for applications where materials are exposed to variable mechanical loads, such as in cutting tools, wear-resistant coatings, and high-stress tribological systems.

**Figure 13:**
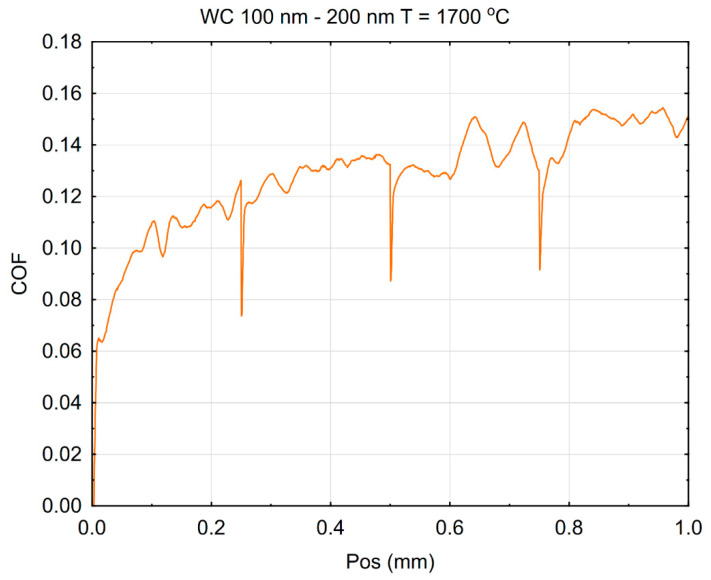
Coefficient of friction (COF) registered during the scratch test of binderless μWC sintered at 1700 °C.

The surface behavior of the tested material can also be assessed by its scratch resistance, which is described using a diagram of friction coefficient in Figure 13.

The achieved friction coefficient values resulted from the sliding properties of the material surface and its resistance to scratching. At the end of the first stage of loading with a normal force 1 N, the friction coefficient was approximately 0.12. An increase in the load at position 0.25 mm caused a short-term drop of COF, which immediately stabilized back close to a value of 0.12 and then rose up to ca. 0.14 before the next increase in the load at position 0.5 mm. At the end of the last stage of loading with 10 N, the coefficient increased slightly to approximately 0.15. This indicates the relatively good resistance of this material’s surface to scratching and deformation under a concentrated load. The pointed element slides across the surface with low resistance and leaves relatively little damage. A higher COF often reflects an increased material–indenter interaction, possibly due to stronger adhesion or more pronounced plastic deformation. Conversely, a lower COF may indicate better surface smoothness, lower adhesion, or formation of a lubricating tribolayer that reduces friction. It also suggests that the binderless μWC material would perform better in applications involving high-contact stresses or abrasive wear, where both surface durability and low friction are critical for long-term performance.

This observation is particularly significant considering the microstructural evolution of the WC material during processing. The initial WC powder had an ultrafine grain size in the range of 100–200 nm. Following consolidation via spark plasma sintering (SPS) at 1700 °C under uniaxial pressure of 50 MPa for 3 min, the grain size increased moderately to 500–900 nm. Although this represents grain growth, the final microstructure remained in the submicron range, which is widely considered optimal for achieving a favorable combination of hardness and fracture resistance in ceramic materials. Typically, the absence of a ductile metallic binder (e.g., Co) in sintered WC can lead to brittle behavior due to limited plastic deformation mechanisms. However, in this study, the sintered WC exhibited unexpected ductile-like response under scratch loading, which can be attributed to several factors: (1) a high relative density and low porosity achieved through the electroconsolidation process, (2) strong intergranular bonding, and (3) the retention of a refined grain structure within the submicron regime. These factors collectively enhance the material’s ability to resist crack initiation and propagation, even without the presence of a metallic phase. This behavior contrasts sharply with that of conventional monolithic ceramics or coarsely sintered WC, where scratch-induced damage is often accompanied by brittle fracture and surface degradation. However, in the case of higher loads, a small so-called “pile-up” effect can be observed [45]. No traces of cohesive damage were found.

While an increase in the sintering temperature caused an increase in the hardness and fracture toughness, the scratch widths and depths were similar for the tested materials. The binderless μWC exhibited stable and uniform plastic deformation without evidence of brittle damage. The plastic deformation observed here confirms the high mechanical reliability of the WC material under contact stress conditions and points to the effectiveness of electroconsolidation as a densification technique.

## 4. Conclusions

The examined binderless μWC sintered using electroconsolidation from the initial powder with a particle size of 100–200 nm, exhibited comparable properties to that of binderless nWC sintered from nanopowders with a particle size of approximately 70 nm. The μWC exhibited hardness of *HV* = 30.06 ± 0.09 GPa, which was higher by almost 14% than that of nWC, but smaller fracture toughness *K_IC_* = 6.59 ± 0.46 MPa·m^1/2^. The hardness increased despite the grains of μWC being ca. 10 times larger than those of nWC. This can be attributed to a more homogeneous microstructure of μWC, with the absence of large agglomerates present in nWC. The binderless WC exhibited lower fracture toughness than the WC–Co systems did, with dominating transgranular fracture close to Griffith cracking. The cracks in composites with cobalt binder exhibited branching and deflections, which contributed to energy dissipation and toughening.

Despite moderate grain growth during sintering, its ductile-like response highlights the potential of a micron-structured WC for use in high-performance applications requiring a balance of extreme hardness, wear resistance, and mechanical robustness in the absence of ductile binders. The binderless μWC material exhibited consistent average property across much of the surface, with small, point-like areas of significant deviations. The demonstrated high hardness makes it a suitable tool material for applications involving weak shock loads. Further investigations including variations in the sintering process, such as longer holding times, may provide ways for further improvement in strength and fracture toughness. In particular, additional actions will be tested to reduce the W_2_C fraction in order to improve fracture toughness with a minimal decrease in hardness. Among other approaches, application of a reducing carbon-rich atmosphere, like CO/CH_4_, during sintering may compensate for carbon loss, while application of carbon-coated WC powders may stabilize stoichiometry during electroconsolidation. A decrease in W_2_C concentration may be expected, which may contribute to an increase in toughness. At the moment, it can be stated that the use of this technology is more cost-effective than using initial tungsten carbide nanopowders, and the provided material is of higher hardness, which, for some applications, can be advantageous.

## Figures and Tables

**Table 1:** Roughness of the sintered specimen.

Measurement No.	After Grinding, Before Polishing		After Polishing	
	Ra, μm	$\mathrm{Arithmetic}\;\mathrm{Mean}\;\bar{Ra}$, μm	Ra, μm	$\mathrm{Arithmetic}\;\mathrm{Mean}\;\bar{Ra}$, μm
1	1.008	1.128	0.007	
2	1.081		0.010	0.010
3	1.294		0.013	

**Table 2:** Quantitative results of the XRD analysis (*T* = 1700 °C, *p* = 45 MPa, holding time of 2 min).

Compound	Powder WC		Sintered Binderless WC		
	Lattice Parameter a	Lattice Parameter c	Content, wt.%	Lattice Parameter a	Lattice Parameter c
WC	2.9038 2.9036	2.8390 2.8387	87.36	2.9063 2.9063	2.8363 2.8363
W_2_C	–	–	12.24	5.1716 5.1715	4.7196 4.7200
C (graphite)	–	–	0.39	2.4612 2.4612	6.7179 6.7265

**Table 3:** Quantitative phase composition of binderless WC samples consolidated at different temperatures.

Sintering Temperature, °C	WC, wt.%	W_2_C, wt.%	C, wt.%	Remarks
1300	100	–	–	Single-phase WC
1400	99.2	0.8	–	Traces of W_2_C detected
1500	97.5	2.0	0.5	Start of decomposition, Equation (3)
1600	92.0	7.0	1.0	Significant W_2_C fraction
1700	87.4	12.2	0.4	Pronounced decomposition

**Table 4:** Results of X-ray spectral microanalysis.

Spectrum No.	16	17	18	19	20
Ca	–	–	2.55	–	–
O	–	–	5.90	1.54	2.08
Na	–	–	2.30	0.70	0.75
Cl	–	–	4.56	8.51	5.68
K	–	–	3.45	8.12	5.23
C	13.96	14.34	30.25	11.11	12.73
W	86.04	85.66	50.98	70.01	73.52
Total	100.00	100.00	100.00	100.00	100.00

**Table 5:** Characteristics of the cemented carbide samples sintered at different parameters from different initial powders.

WC Sintered Samples	Sintering Parameters		Relative Density, %	Grain Size, μm	Hardness *HV*_5_, GPa	Fracture Toughness *K_IC_*, MPa∙m^0.5^	Reference
	**Temperature, °C**	**Time, min**					
nWC	1750	1	99.2	0.1	26.4 ± 0.5	8.5 ± 0.5	[18]
WC–8 wt.%Co	1450	60	99.0	3–4	16 ± 0.5	12 ± 0.5	[18]
WC–6 wt.%Co	1350	3	not reported	5–6	15.5 ± 0.2	9.0 ± 0.4	[36]
μWC	1700	3	99.0	0.8–1	30 ± 0.09	6.59 ± 0.46	This work

## Data Availability

The original contributions presented in this study are included in the article. Further inquiries can be directed to the corresponding authors.
